# LncRNA PITPNA‐AS1 boosts the proliferation and migration of lung squamous cell carcinoma cells by recruiting TAF15 to stabilize HMGB3 mRNA

**DOI:** 10.1002/cam4.3268

**Published:** 2020-09-01

**Authors:** Ping Ren, Lei Xing, Xiaodong Hong, Liang Chang, Hong Zhang

**Affiliations:** ^1^ Department of Thoracic Surgery The First Hospital of Jilin University Changchun P.R. China

**Keywords:** HMGB3, lung squamous cell carcinoma (LUSC), PITPNA antisense RNA 1 (PITPA‐AS1), TAF15

## Abstract

Plenty of reports have probed the involvement of abnormally expressed lncRNAs in multiple cancers, including lung squamous cell carcinoma (LUSC). Through online database GEPIA, lncRNA PITPNA antisense RNA 1 (PITPNA‐AS1) was highly expressed in LUSC samples, and these tendency was further affirmed in LUSC cells. The aim of current study was to investigate the related mechanism of PITPNA‐AS1 in LUSC. Functional experiments verified that depletion of PITPNA‐AS1 hampered the proliferative and migratory abilities, but accelerated apoptosis of LUSC cells. Additionally, we observed the increased expression of HMGB3 and its positive correlation with PITPNA‐AS1 in LUSC samples. Interestingly, PITPNA‐AS1 mainly located in the cytosol of LUSC cells, and also affected mRNA stability of HMGB3. Furthermore, the repressed mRNA stability of HMGB3 by PITPNA‐AS1 via TAF15 was exposed through mechanism experiments. The mediatory function of PITPNA‐AS1 on HMGB3 was validated via rescue assays. All in all, PITPNA‐AS1 promoted the proliferation and migration of LUSC cells via stabilizing HMGB3 by TAF15. In conclusion, our study displayed a novel mechanism underlying PITPNA‐AS1 in LUSC cells.

## Graphical abstract

PITPNA‐AS1 is dramatically elevated and promotive in LUSC cells. PITPNA‐AS1 positively affects HMGB3 in LUSC cells. PITPNA‐AS1 and TAF15 mediates HMGB3 synergistically.

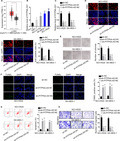

## 1 INTRODUCTION

Lung cancer is the major cause of cancer‐related deaths globally, which is divided into several different subtypes.^1^, ^2^ Lung squamous cell carcinoma (LUSC) takes up around 80% of non‐small‐cell lung cancer (NSCLC), a commonest type of lung cancer.^3^, ^4^ LUSC frequently happens in older males along with high metastasis and recurrence rates.^5^ Regardless of the prior treatment of chemotherapy, molecular‐targeted therapy or radiotherapy, the prognosis of LUSC patients remains dismal. Thus, searching for promising targets is necessary for improvement of LUSC treatment.

Various reports have proved the functions of long non‐coding RNAs (lncRNAs) in human cancers. For example, lncRNA GHET1 predicts unfavorable prognosis of hepatocellular carcinoma patients and boosts proliferation via silencing of KLF2.^6^ HOXA11‐AS enhances proliferative HCC cells through epigenetic silencing of DUSP5.^7^ LncRNA TUG1 potentiates the proliferation and apoptosis resistance via AURKA in epithelial ovarian cancer.^8^ LncRNA PITPNA antisense RNA 1 (PITPNA‐AS1), localized in chromosome 17p13.3 and not reported in LUSC before, which was assumed to be highly expressed in LUSC based on online database. Therefore, this study focused on the mechanism underneath PITPNA‐AS1 functions in LUSC.

RNA‐binding proteins (RBPs) are known to exert their functions via interplaying with RNAs to affect stability, expression, function, and cellular location in tumors.^9^, ^10^ For instance, LINC01232 exerts promotive properties in pancreatic adenocarcinoma upon TM9SF2 by recruiting EIF4A3.^11^ LIN28B‐AS1 activates LIN28B by binding to IGF2BP1 in lung adenocarcinoma.^12^ LncRNA HCG22 represses the proliferation and metastasis of bladder cancer cells via PTBP1.^13^ TATA‐box binding protein associated factor 15 (TAF15) belongs to the TET family of RBPs. Although a previous study has explained the tumor‐specific role of TAF15,^14^ more investigations of TAF15 in human cancers are requisite.

High mobility group box 3 (HMGB3) is an identified oncogene in the development of human cancers. For example, HMGB3 is negatively modulated by miR‐200b/c‐3p and facilitates the proliferation and metastasis of glioblastoma cells.^15^ HMGB3 enhances growth and migration of colorectal carcinoma cells via Wnt/β‐catenin pathway.^16^ MiR‐200b restrains hepatocellular carcinoma proliferation as well as migration by reducing HMGB3.^17^ The carcinogenic function of HMGB3 in lung cancer has also been proven.^18^, ^19^ The precise mechanism in the upstream of HMGB3 in LUSC remains to be investigated.

To summarize, our current study focused on the expression profile and functions of PITPNA‐AS1 in LUSC cells and also probed the correlation between PITPNA‐AS1 and HMGB3 in LUSC.

## 2 MATERIALS AND METHODS

### 2.1 Cell lines and reagent

LUSC cells (EBC‐1, NCI‐H520, NCI‐H1703, SK‐MES‐1) and normal HLF‐a cells were both procured from ATCC (Manassas, VA) and maintained in the DMEM (Invitrogen, Carlsbad, CA) with 95% air and 5% CO_2_ at 37℃. One per cent antibiotics solution and 10% FBS (Invitrogen) served as the medium supplements. In addition, the Actinomycin D (2 μg/mL) was commercially acquired from Sigma‐Aldrich (St. Louis, MO) to treat NCI‐H520 and SK‐MES‐1 cells.

### 2.2 qRT‐PCR analysis

Total RNA extracts were acquired by TRIzol reagent (Invitrogen) from NCI‐H520, SK‐MES‐1, and EBC‐1 cell samples, then converted into cDNA. qPCR was performed in line with the manual of one Step SYBR^®^ PrimeScript™ RT‐PCR Kit (Perfect Real Time; Takara, Shiga, Japan). Relative gene expression was calculated as per the 2^‐ΔΔCt^ method.

### 2.3 Transfection

The shRNAs targeting PITPNA‐AS1 (sh‐PITPNA‐AS1#1/2), TAF15 as well as other genes (DDX54, DGCR8, FMR1, IGF2BP1, IGF2BP2, LIN28B, SRSF1) were utilized for gene silencing, with negative control (NC) shRNAs (sh‐NC). The pc‐PITPNA‐AS1, pc‐TAF15, pc‐HMGB3, and empty pcDNA3.1 vector was synthesized for gene overexpression. All these plasmids were produced by Genepharma (Shanghai, China). Transfection kit Lipofectamine 2000 (Invitrogen) was used to transfect into cells for 48 hours.

### 2.4 EdU assay

After transfection, LUSC cells seeded in the 96‐well plates were subjected to EdU assay kit (Ribobio, Guangzhou, China) for 3 hours, then treated with 4% paraformaldehyde, 0.5% Troxin X‐100. After DAPI staining for cell nuclei, EdU‐positive cells were observed by fluorescence microscopy (Olympus, Tokyo, Japan). Each procedure was repeated at least in triplicate.

### 2.5 Colony formation assay

To assess the colony‐forming efficiency of LUSC cells, transfected cells were planted at 500 cells/well into the six‐well plates, grown in complete media containing 10% FBS. After incubated for 2 weeks, which cells were fixed with 4% paraformaldehyde and stained with 0.5% crystal violet. Colonies were counted manually. Each procedure was repeated at least in triplicate.

### 2.6 TUNEL assay

TUNEL assay was performed in LUSC cells based on the protocol of In Situ Cell Death Detection Kit (Roche, Basel, Switzerland). Transfected cells were rinsed and cultured in the six‐well plates for fixing and staining. The TUNEL‐positive cells were counted under fluorescence microscopy. Each procedure was repeated at least in triplicate.

### 2.7 Flow cytometry for apoptosis

Transfected cells were collected and washed with PBS. Cells were collected and fixed in 70% cold ethanol for 1 hour, then double‐stained with Annexin V‐fluorescein isothiocyanate (FITC) and propidium iodide (PI) in the dark. Cell apoptosis was analyzed by flow cytometry (BD Biosciences, San Jose, CA). Each procedure was repeated at least in triplicate.

### 2.8 Transwell migration assay

Transfected cells (2 × 10^4^ cells per well) in serum‐free medium were plated into the upper chamber of the 8 μm pore transwell apparatus (Corning Incorporated, Corning, NY), while lower chamber was added with the complete medium. 24 hours later, migrated cells were fixed and stained with 0.5% crystal violet.

### 2.9 FISH assay

FISH assay was performed when the cell confluence reached to 60%‐70%. After permeabilization, cells were incubated with the FISH probe designed for PITPNA‐AS1 (Ribobio) in the hybridization buffer overnight. Following counterstaining with DAPI and washing with PBS for three times, cells were photographed with fluorescence microscopy.

### 2.10 Subcellular fractionation

Nuclear and cytoplasmic RNAs were severally isolated from cultured LUSC cell samples according to the instruction of PARIS™ Kit (Invitrogen). qRT‐PCR was employed to determine the RNA (GAPDH, U6, PITPNA‐AS1) levels in fractions.

### 2.11 Luciferase reporter assay

HMGB3 promoter were cloned into the upstream of firefly luciferase and inserted into the downstream of pGL3 reporter plasmids (Promega, Madison, WI, USA). To evaluate the luciferase activity, HMGB3 promoter vector was co‐transfected into LUSC cells with sh‐NC or sh‐PITPNA‐AS1#1. NCI‐H520 and SK‐MES‐1 cells were seeded at 8000 cells/well and co‐transfected with indicated transfection plasmids and pRL‐TK‐Renilla plasmid (Promega) using Lipofectamine 2000 reagent (Thermo Fisher Scientific). Forty‐eight hours later, relative luciferase activity was estimated. Each procedure was repeated at least in triplicate.

### 2.12 RNA pull‐down assay

The protein extracts obtained from LUSC cells were reaped for incubation with the1 μM of biotinylated PITPNA‐AS1‐WT or PITPNA‐AS1‐Mut probe (or biotinylated HMGB3‐WT or HMGB3‐Mut) were incubated with lysates obtained from 1 × 10^7^ NCI‐H520 or SK‐MES‐1 cells for 1 hours. After that, streptavidin agarose magnetic beads were added to isolate the RNA‐protein complex. Finally, the complexes were then analyzed by western blot analysis.

### 2.13 RIP assay

RIP assay was conducted as described previously.^20^ Cells (1 × 10^7^) were lysed in RIP buffer that was supplemented with RNase A inhibitor and DNase I before centrifugation. Cell lysates were incubated with protein A/G beads coated with the antibodies against TAF15 and control IgG at 4°C for 3 hours. After washing, the immunocomplexes were eluted using elution buffer containing 50 mM Tris [pH 8.0], 1% SDS, and 10 mM EDTA at 65°C for 10 min. To isolate RNAs, samples were treated with proteinase K. Following RNA isolation and purification, Purified RNAs were then subjected to RT‐PCR analysis.

### 2.14 Western Blot

Cellular protein extracts from NCI‐H520, SK‐MES‐1, and EBC‐1 cell samples were acquired for electrophoresis on 12% SDS‐PAGE gel, then shifted to PVDF membranes and treated with 5% skimmed milk. Primary antibodies against GAPDH, HMGB3, TAF15, and appropriate secondary antibodies tagged with HRP were obtained from Abcam (Cambridge, MA) and utilized after dilution. Band density was analyzed by the enhanced chemiluminescence reagent (Santa Cruz Biotechnology, Santa Cruz, CA).

### 2.15 Statistical analysis

More than three biological repeats were included for each experiment, and the results were all given as the mean ± SD. PRISM 6 (GraphPad, San Diego, CA) was applied to analyze data through Student's t‐test or one‐way ANOVA, with *P* < .05 as the significant level.

## 3 RESULTS

### 3.1 PITPNA‐AS1 level was dramatically elevated in LUSC cells

**Figure 1:**
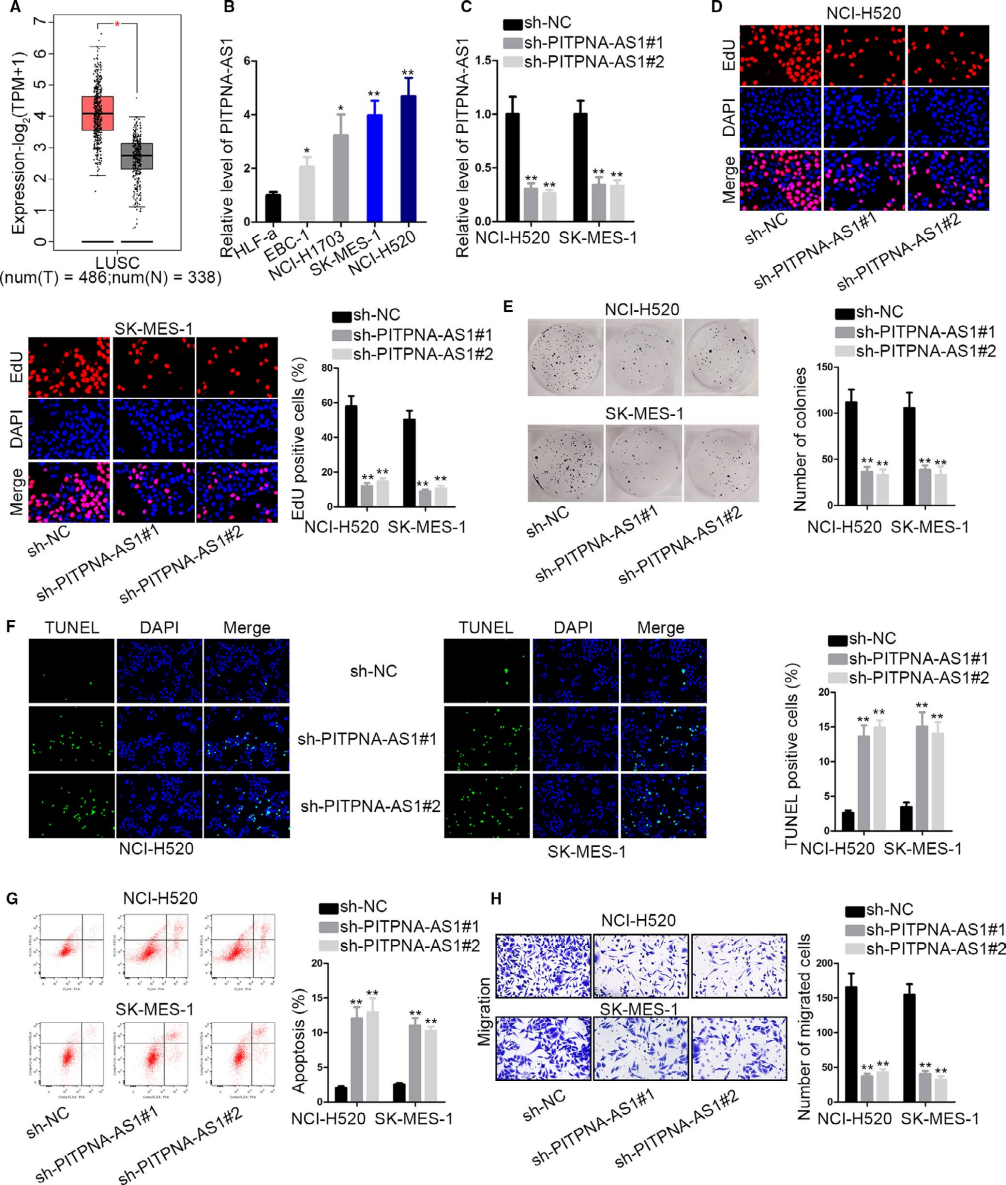
PITPNA‐AS1 was upregulated in LUSC cells. (A) GEPIA prediction of PITPNA‐AS1 level in LUSC. (B) Usage of qRT‐PCR for PITPNA‐AS1 level in LUSC cells comprising EBC‐1, NCI‐H1703, SK‐MES‐1, and NCI‐H520, as well as normal cell line HLF‐a. (C) Usage of qRT‐PCR for PITPNA‐AS1 level in sh‐PITPNA‐AS1#1/2‐transfected NCI‐H520 and SK‐MES‐1 cells. (D‐E) Cell proliferation detection by EdU staining and colony formation experiments in two cells with PITPNA‐AS1 knockdown. (F‐G) Cell apoptosis evaluation by TUNEL staining and flow cytometry analysis in PITPNA‐AS1‐silenced cells. (H) Transwell assay detected cell migration ability of two transfected cells. ^*^ *P* < .05 and ^**^ *P* < .01 vs controls

Through GEPIA (http://gepia2.cancer‐pku.cn/#index) dataset, we identified the high level of PITPNA‐AS1 in LUSC tissues (Figure 1A). After detection of qRT‐PCR, PITPNA‐AS1 was proved to be high in four LUSC cells in contrast to normal cells HLF‐a (Figure 1B). Thus, we further evaluated the function of PITPNA‐AS1 in LUSC cells. Before functional assays, we silenced PITPNA‐AS1 in NCI‐H520 and SK‐MES‐1 cells with highest PITPNA‐AS1 expression to investigate the role of PITPNA‐AS1 in LUSC (Figure 1C). The transfection efficiency obtained from flow cytometry has been shown in Supplementary file. EdU staining illustrated that silencing of PITPNA‐AS1 lead to reduced EdU‐stained cells (Figure 1D). Colony‐forming assay demonstrated that decreased colony‐forming numbers of two cells under transfection of sh‐PITPNA‐AS1#1/2 (Figure 1E). These two experiments exhibited that cell proliferation was inhibited when PITPNA‐AS1 was knocked down. As for cell apoptosis, TUNEL staining and flow cytometry analysis were conducted in indicated LUSC cells. The apoptosis rate was higher in sh‐PITPNA‐AS1#1/2 group than those in sh‐NC group (Figure 1F‐G). In transwell migration assay, migrated cells were lowered after treated with sh‐PITPNA‐AS1#1/2 (Figure 1H). These findings elucidated that PITPNA‐AS1 was expressed at high level in LUSC cells and had oncogenic property.

### 3.2 PITPNA‐AS1 positively regulated HMGB3 expression in the cytoplasm of lusc cells

**Figure 2:**
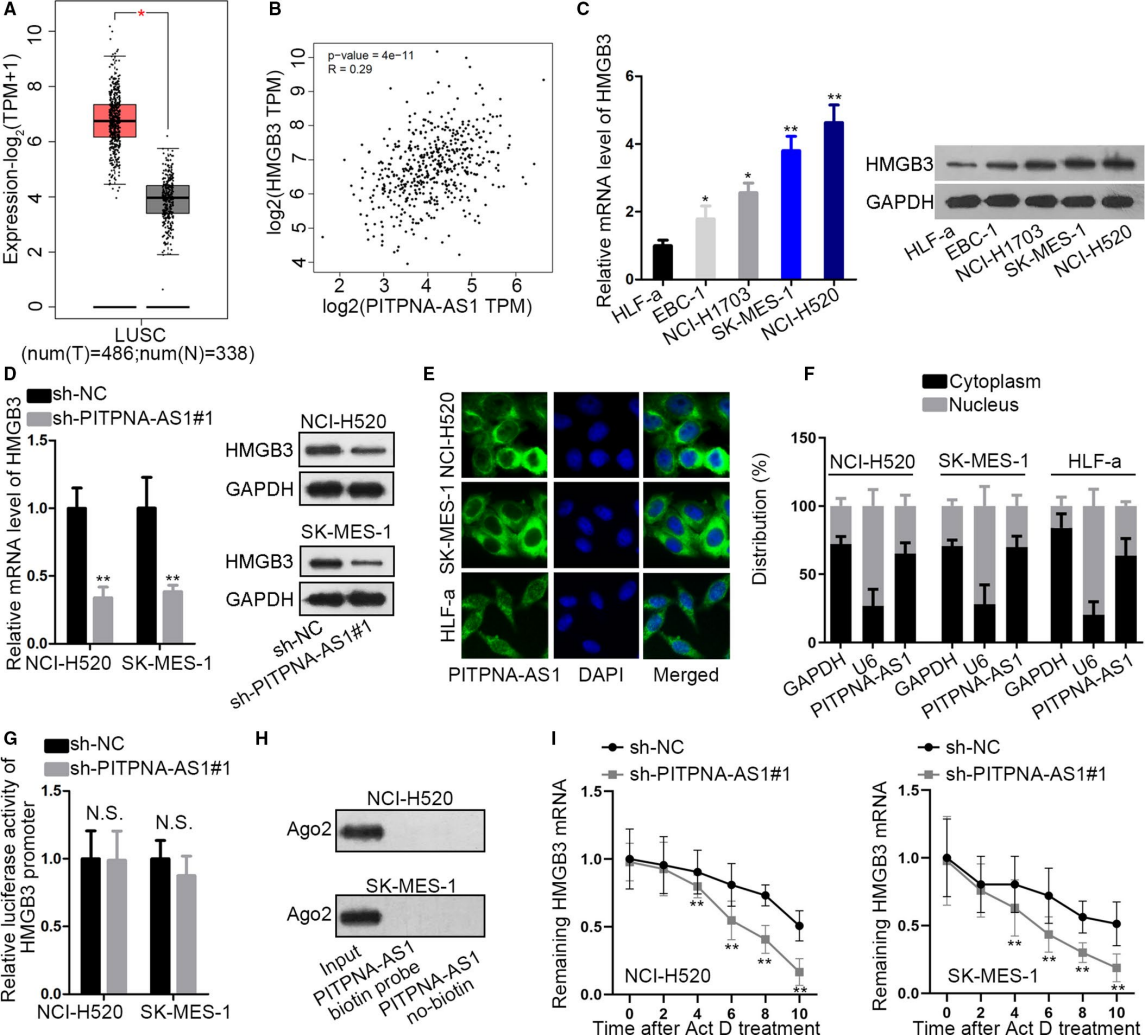
PITPNA‐AS1 positively regulated HMGB3 expression. (A‐B) GEPIA prediction of HMGB3 level and its correlation with PITPNA‐AS1 in LUSC. (C) HMGB3 expression at mRNA level and protein level in four tumor cells and one normal cell line, as determined via qRT‐PCR and western blotting. (D) Application of qRT‐PCR and western blotting for HMGB3 influenced by sh‐PITPNA‐AS1#1. (E‐F) The position of PITPNA‐AS1 was explored through subcellular fractionation and FISH experiments. (G) Luciferase reporter assay with HMGB3 promoter in NCI‐H520 and SK‐MES‐1 cells treated with sh‐NC or sh‐PITPNA‐AS1#1. (H) RIP assay tested whether PITPNA‐AS1 was precipitated via anti‐Ago2 in two cells. (I) qRT‐PCR after Act D treatment for determining the half‐life of HMGB3 under PITPNA‐AS1 silence. ^*^ *P* < .05 and ^**^ *P* < .01 vs controls. NS means not significant

Since lncRNAs can mediate mRNAs to affect cellular activities of tumors, we continued to browse GEPIA dataset and figured out HMGB3 is a highly expressed mRNA in GEPIA LUSC samples (Figure 2A). Importantly, HMGB3 had a positive expression correlation with PITPNA‐AS1 (Figure 2B). HMGB3 expression in LUSC cells and normal cells was tested at mRNA level and protein level. The data presented that the levels of HMGB3 mRNA and protein were overtly augmented in LUSC cells compared with control cells (Figure 2C). Additionally, the negative effect of PITPNA‐AS1 silence on HMGB3 expression was validated through qRT‐PCR and western blotting (Figure 2D). Thereafter, we aimed to excavate the related mechanism between PITPNA‐AS1 and HMGB3. Subcellular fractionation and FISH assays determined the main distribution of PITPNA‐AS1 in the cytosol of LUSC cells and normal HLF‐a cell (Figure 2E‐F). Luciferase reporter assay demonstrated that HMGB3 promoter activity was unaffected by the knockdown of PITPNA‐AS1 (Figure 2G). Cytoplasmic lncRNAs can exert functions through serving as competing endogenous RNAs (ceRNAs). Through RNA pull‐down assay, we discovered that PITPNA‐AS1 was not immunoprecipitated in anti‐Ago2 group (Figure 2H), excluding ceRNA mechanism of PITPNA‐AS1. Additionally, cytoplasmic lncRNAs can mediate mRNA stability. qRT‐PCR with Act D treatment disclosed that the half‐life of HMGB3 mRNA was obviously shortened via PITPNA‐AS1 down‐regulation (Figure 2I). Taken together, PITPNA‐AS1‐mediated HMGB3 expression at post‐transcriptional level.

### 3.3 TAF15 served as the RBP of HMGB3

**Figure 3:**
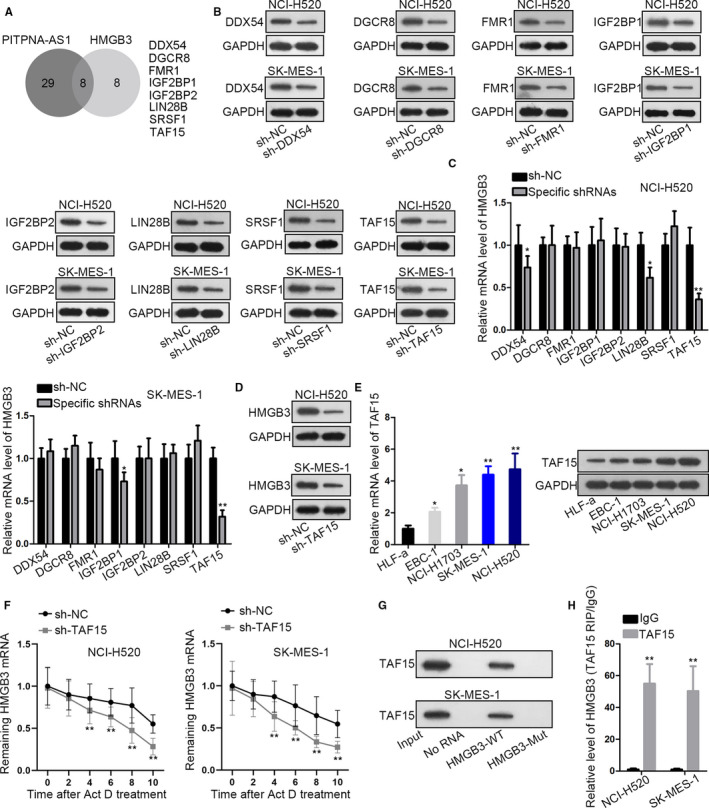
TAF15 acted as the RBP to stabilize HMGB3. (A) Venn diagram of eight RBPs binding to PITPNA‐AS1 or HMGB3. (B) Western blotting was utilized for measuring transfection efficacies. (C) HMGB3 mRNA level under different transfections in NCI‐H520 and SK‐MES‐1 cells. (D) HMGB3 protein level in two cells under treatment of sh‐TAF15. (E) TAF15 mRNA and protein levels in five cells were analyzed via qRT‐PCR and western blotting. (F) qRT‐PCR determined HMGB3 half‐life under TAF15 silence post Act D treatment. (G‐H) RNA pull‐down and RIP experiments were adopted to test the interplay between TAF15 and HMGB3. ^*^ *P* < .05 and ^**^ *P* < .01 vs controls

To discover the proteins worked between PITPNA‐AS1 and HMGB3 in LUSC, we searched on starBase v3.0. Eight proteins were disclosed to bind with both PITPNA‐AS1 and HMGB3 (Figure 3A). Among these RBPs, TAF15 is selected for investigation as TAF15 silencing distinctly decreased HMGB3 level (Figure 3B‐D). Both mRNA and protein levels of TAF15 were higher in LUSC cells (Figure 3E). To determine the influence of TAF15 on HMGB3 stability, we analyzed the mRNA expression of HMGB3 under Act D treatment. We discovered that HMGB3 mRNA half‐life was distinctly shortened through silencing of TAF15 (Figure 3F). The interaction between HMGB3 and TAF15 was verified through RIP ad RNA pull‐down experiments. TAF15 protein was merely pulled down by biotinylated HMGB3‐WT probe and HMGB3 was abundantly precipitated by anti‐TAF15 (Figure 3G‐H). These results elucidated that TAF15 stabilized HMGB3 mRNA.

### 3.4 PITPNA‐AS1 and TAF15 synergistically mediated HMGB3 stability

**Figure 4:**
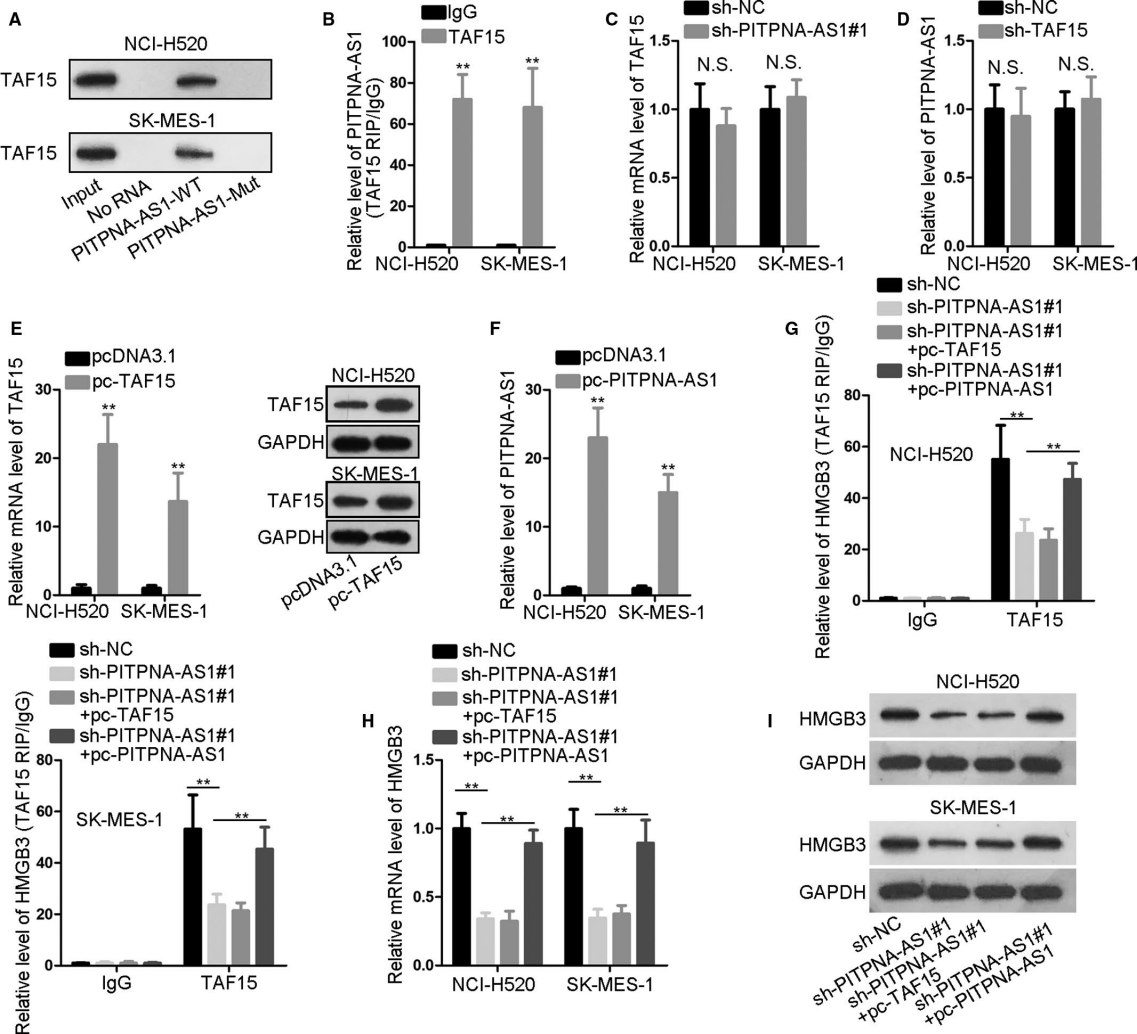
PITPNA‐AS1 and TAF15 synergistically regulated HMGB3. (A‐B) The binding of TAF15 to PITPNA‐AS1 was affirmed through RNA pull‐down and RIP experiments. (C‐D) Expression of TAF15 and PITPNA‐AS1 under specific treatment by qRT‐PCR. (E‐F) The overexpression efficiency for TAF15 or PITPNA‐AS1 was estimated via qRT‐PCR or western blotting. (G) RIP assay was employed to evaluate the binding affinity between TAF15 and HMGB3 in four groups. (H‐I) Analyses of qRT‐PCR and western blotting for HMGB3 in NCI‐H520 and SK‐MES‐1 cells of four groups. ^**^ *P* < .01 vs controls. NS means not significant

Further, we explored the interplay of PITPNA‐AS1 with TAF15. RNA pull‐down and RIP experiments affirmed the binding of PITPNA‐AS1 with TAF15. It was found that TAF15 was enriched in the complex pulled down by bio‐PITPNA‐AS1‐WT probe and PITPNA‐AS1 was largely precipitated by anti‐TAF15 (Figure 4A‐B). Also, we found out that there was no expression change of TAF15 or PITPNA‐AS1 in response to knockdown of PITPNA‐AS1 or TAF15 in two LUSC cells (Figure 4C‐D). Considering that RBPs are recruited by lncRNAs to develop their functions, we suspected that PITPNA‐AS1 might affect HMGB3 through recruiting TAF15. In EBC‐1 cells, TAF15 and PITPNA‐AS1 were separately up‐regulated after transfections (Figure S1A‐B). The interaction between TAF15 and HMGB3 was not changed through addition of PITPNA‐AS1 or TAF15, but overtly promoted via up‐regulation of PITPNA‐AS1 and TAF15 (Figure S1C). Similarly, the mRNA and protein levels of HMGB3 in EBC‐1 cells under transfection of pc‐TAF15 or pc‐PITPNA‐AS1 were not changed while increased post co‐transfection of pc‐TAF15 + pc‐PITPNA‐AS1 (Figure S1D). Here, we affirmed the PITPNA‐AS1‐TAF15 axis regulated HMGB3. In subsequence, TAF15 and PITPNA‐AS1 were separately overexpressed in NCI‐H520 and SK‐MES‐1 cells (Figure 4E‐F). The transfection efficiency obtained from flow cytometry has been shown in Supplementary file. RIP assay proved that the interplay between TAF15 and HMGB3 was refrained via PITPNA‐AS1 depletion but recovered via addition of PITPNA‐AS1, but not by that of TAF15 (Figure 4G). The expression of HMGB3 showed the same trend (Figure 4H‐I). Based on these data, we concluded that PITPNA‐AS1 and TAF15 synergistically promoted HMGB3 expression.

### 3.5 Overexpression of HMGB3 reversed the repressive impact of pitpna‐AS1 knockdown on lusc cells

**Figure 5:**
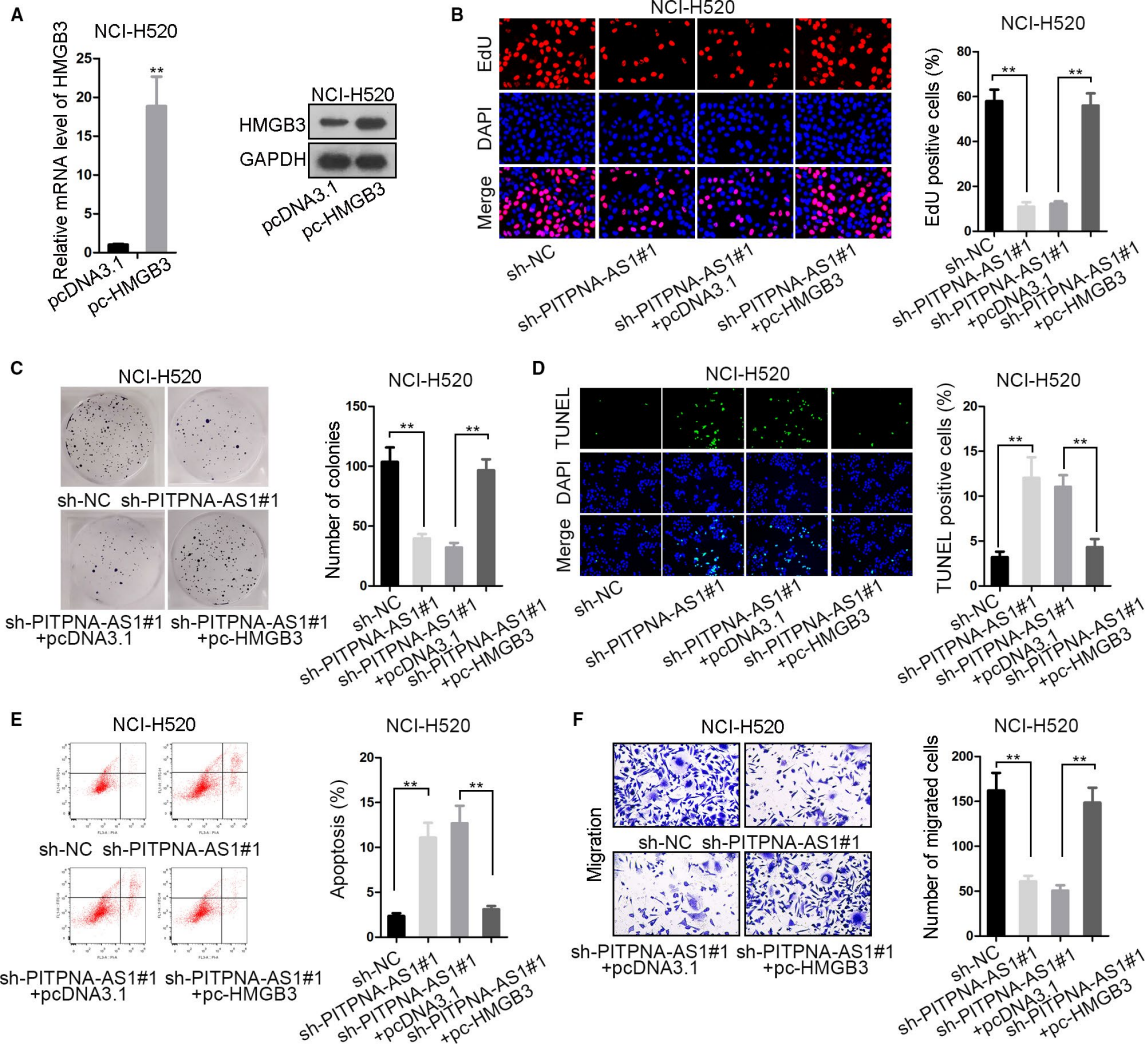
Overexpression of HMGB3 reversed the impact of PITPNA‐AS1 knockdown on LUSC cells. (A) HMGB3 was overexpressed and the efficiency was assessed via qRT‐PCR and western blot. (B‐C) EdU staining and colony formation experiments estimated the proliferative capability of NCI‐H520 cells in four various groups. (D‐E) TUNEL staining and flow cytometry analysis assessed the apoptosis in differently treated cells. (F) Transwell assay detected the migratory ability in four groups of cells. ^**^ *P* < .01 vs controls

For rescue assays, HMGB3 was up‐regulated via transfecting pc‐HMGB3 into NCI‐H520 cells (Figure 5A). The transfection efficiency obtained from flow cytometry has been shown in Supplementary file. The reduced EdU positive cells in sh‐PITPNA‐AS1#1‐transfected NCI‐H520 cells were raised when HMGB3 expression vector was transfected into (Figure 5B). Moreover the declined colonies by repression of PITPNA‐AS1 were increased after HMGB3 overexpression (Figure 5C). Here, cell proliferation was restrained through down‐regulation of PITPNA‐AS1 but recovered with overexpression of HMGB3. TUNEL staining disclosed that cell apoptosis was accelerated by silenced PITPNA‐AS1, which was abolished by up‐regulated HMGB3 (Figure 5D). This result was further certified by flow cytometry analysis (Figure 5E). As for cell migration, transwell assay demonstrated that the lowered migrated cells under transfection of sh‐PITPNA‐AS1#1 were abrogated when HMGB3 level was increased (Figure 5F). All in all, PITPNA‐AS1 modulated HMGB3 to affect LUSC cell proliferation and migration.

## 4 DISCUSSION

In our research, lncRNA PITPNA antisense RNA 1 (PITPNA‐AS1) was functionally characterized and facilitated the proliferation and migration of LUSC cells. PITPNA‐AS1 recruited TAF15 to stabilize HMGB3 in LUSC cells.

It is increasingly known that long non‐coding RNAs (lncRNAs) are dominant effectors in tumors.^21^ GEPIA database presented the increased level of PITPNA‐AS1 in LUSC, which prompted us to explore the possible role of PITPNA‐AS1 in LUSC. To the best of our knowledge, there was no previous study reported the role of PITPNA‐AS1 in LUSC. Our work unveiled that PITPNA‐AS1 expression was relatively elevated in LUSC cells. Subsequent functional assays testified that downregulation of PITPNA‐AS1 hampered cell and migration, but enhanced apoptosis rate in LUSC cells. This study was the first time to unveil the expression and function of PITPNA‐AS1 in LUSC.

LncRNAs can regulate their downstream mRNAs,^22^, ^23^ we sought to search for PITPNA‐AS1‐associated genes in LUSC. High mobility group box 3 (HMGB3), an up‐regulated gene with significantly positive relationship with PITPNA‐AS1 in LUSC samples. Recent papers have elucidated the tumor‐promoting role of HMGB3 in various ranges of cancers.^16^, ^17^, ^18^ Thus, we investigated the mechanism of PITPNA‐AS1 in regulating HMGB3. It was uncovered that PITPNA‐AS1 was located in the cytoplasm of LUSC cells, implying that PITPNA‐AS1 potentially regulated HMGB3 at post‐transcriptional level. Cytoplasmic lncRNAs are known as ceRNAs in human cancers.^24^, ^25^, ^26^ Here, we found that PITPNA‐AS1 could not be pulled down by anti‐Ago2, excluding the ceRNA mechanism of PITPNA‐AS1 in LUSC. Previous papers have discussed the existence of lncRNAs between RBPs and downstream genes. For instance, ZEB1‐activated LBX2‐AS1 interplays with HNRNPC to strengthen the stability of ZEB1 and ZEB2 and promotes esophageal squamous cell carcinoma.^27^ LncRNA B4GALT1‐AS1 recruiting HuR promotes osteosarcoma cells migration and stemness via stimulating YAP.^28^ In this study, we browsed starBase v3.0 to gain the functional RNA‐binding protein (RBP) interplaying with PITPNA‐AS1 or HMGB3. TATA‐box binding protein associated factor 15 (TAF15) was chosen after screening. Mechanism experiments testified the binding of TAF15 to PITPNA‐AS1 or HMGB3, which further concluded that PITPNA‐AS1 recruited TAF15 to synergistically promote HMGB3 in LUSC. Therefore, we confirmed that PITPNA‐AS1 recruited TAF15 to HMGB3 3’UTR, thus stabilizing HMGB3 mRNA.

In conclusion, our research revealed the expression pattern and functions of PITPNA‐AS1 in LUSC cells. More importantly, PITPNA‐AS1/TAF/HMGB3 was found to be a novel molecular pathway contributing to LUSC progression. All our findings may provide novel potential therapeutic target for LUSC.

## CONFLICTS OF INTEREST

None.

## AUTHOR CONTRIBUTION

Ping Ren designed this study and was responsible for article writing and prepared all figures. Lei Xing and Xiaodong Hong collected experimental materials. While Liang Chang and Hong Zhang recorded and analyzed experimental data. All authors have made substantial contributions to this study.

## ETHICAL APPROVAL

Not applicable.

## Supporting information

Figure S1.

Supplementary Material

## Data Availability

Research data are not shared.
