# Supplementary figures and images for: LncRNA PITPNA‐AS1 boosts the proliferation and migration of lung squamous cell carcinoma cells by recruiting TAF15 to stabilize HMGB3 mRNA
Source: Cancer Med. 2020 Sep 1;9(20):7706–16. doi: 10.1002/cam4.3268 (PMC7571819; doi:10.1002/cam4.3268)

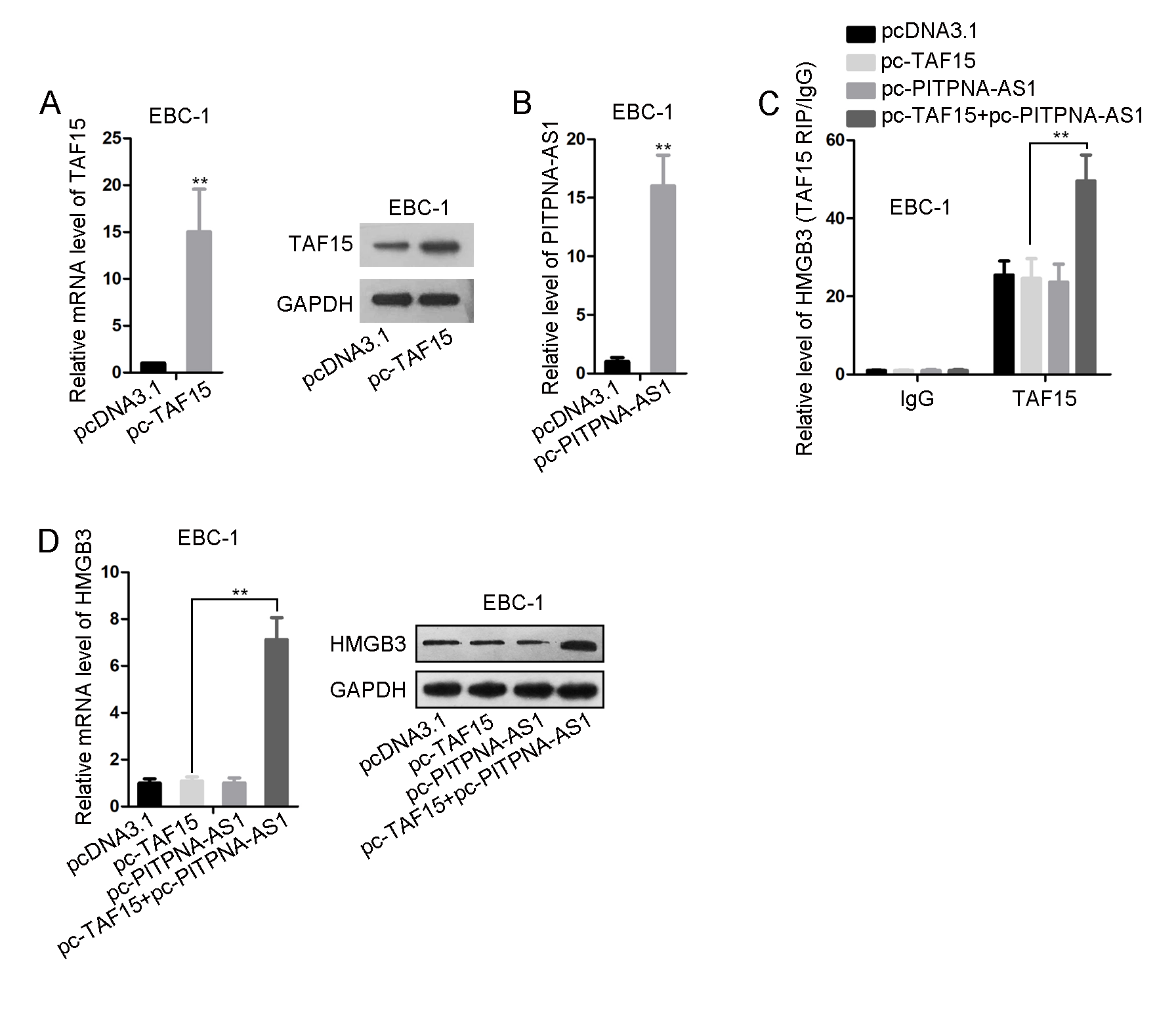

Supplement: Supplementary file 1 — Figure S1. [file CAM4-9-7706-s001.tif]

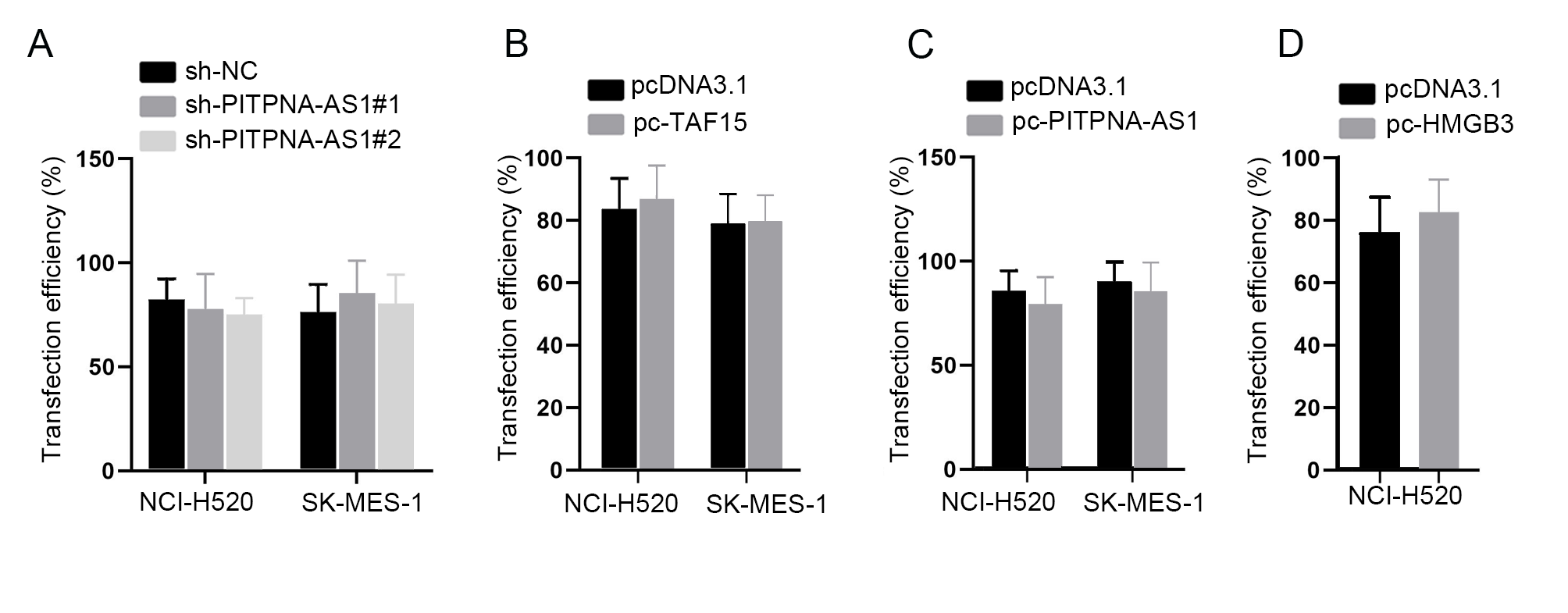

Supplement: Supplementary file 2 — Supplementary Material [file CAM4-9-7706-s002.tif]
